# Possible Efficacy of Vaginal Progesterone on Asymptomatic Women with a Short Cervix after 24 Weeks of Gestation: A Historical Cohort Study in Japan

**DOI:** 10.31662/jmaj.2024-0036

**Published:** 2024-09-20

**Authors:** Naoki Otsuka, Kenji Imai, Sho Tano, Seiko Matsuo, Takafumi Ushida, Masataka Nomoto, Yukako Iitani, Mika Ishi, Yosuke Kawai, Toshimitsu Furui, Hiroaki Kajiyama, Tomomi Kotani

**Affiliations:** 1 Division of Obstetrics and Gynecology, Ogaki Municipal Hospital, Gifu, Japan; 2 Department of Obstetrics and Gynecology, Nagoya University Graduate School of Medicine, Nagoya, Japan

**Keywords:** Progesterone, Preterm labor, Short cervix, Cervical insufficiency

## Abstract

**Introduction:**

Few studies have explored the preventive efficacy of vaginal progesterone (VD) treatment for preterm delivery (PTD) in Japanese clinical practice. In this study, the efficacy of the VD treatment in pregnant women with a short cervix (sCX) diagnosed after 24 weeks is evaluated, focusing on perinatal outcomes.

**Methods:**

A retrospective historical cohort study. Clinical data of 273 singleton women hospitalized for preventing PTD were extracted. Inclusion criteria are diagnosed sCX at 24-33 weeks. We excluded women with factors including treatment start before 24 weeks, medically induced PTD, PTD on admission day, and fetal demise. Consequently, logistic regression analyses were conducted on data from 79 women during Period 1 (November 2015 to March 2018, using prolonged intravenous ritodrine hydrochloride) and 82 women during Period 2 (August 2018 to August 2022, implementing VD treatment), adjusting maternal age, parity, body mass index, gestational age, cervical length, and histological chorioamnionitis. The primary outcomes involved PTD <37 and <34 weeks and neonatal intensive care unit admission. Secondary outcomes included the interval from the diagnosis of sCX to delivery <14 and <28 days, infant intubation, and surfactant administration. Since VD use is off-label in Japan, we obtained written informed consent prior to treatment.

**Results:**

VD treatment (Period 2) significantly decreased the incidence of PTD (birth < 37 weeks) (adjusted odds ratios [ORs] 0.43, 95% confidence intervals [CIs] 0.19-0.96), impending delivery within 14 and 28 days after confirming sCX (adjusted OR 0.12, 95% CI 0.06-0.72; adjusted OR 0.25, 95% CI 0.09-0.74, respectively), and neonatal intubation rate (adjusted OR 0.17, 95% CI 0.04-0.75).

**Conclusions:**

The VD treatment can prevent PTD in asymptomatic women with sCX diagnosed after 24 weeks of gestation. Although further validation is warranted, these findings may contribute to expanding the use of VD treatment in Japanese clinical practice.

## Introduction

Even though there have been numerous efforts to understand and mitigate its risk factors, preterm delivery (PTD) is still one of the most challenging problems in modern obstetrics and the primary cause of adverse outcomes in newborns ^(1), (2), (3)^. In predicting spontaneous PTD, the measurement of cervical length (CL) in the early second trimester (i.e., <24 weeks of gestation) is the most powerful predictor ^(4), (5)^. Several studies have also indicated that short CL measured after 24 weeks of gestation is a substantial risk factor for spontaneous PTD ^(4), (6)^. In terms of risk mitigation, the efficacy of vaginal progesterone treatment has been repeatedly demonstrated and recommended for asymptomatic pregnant women with a short cervix before 24 weeks ^(7), (8), (9), (10)^. Additionally, several randomized controlled trial (RCT) studies have demonstrated that vaginal progesterone treatment initiated after 24 weeks of gestation could be beneficial for PTD prevention ^(11), (12), (13)^. Nonetheless, the progesterone treatment has not been approved at present by the Japan Ministry of Health, Labor, and Welfare, and no study has evaluated the preventive efficacy of the progesterone treatment initiated after 24 weeks of gestation in Japanese clinical practice.

In the traditional approach to PTD prevention in Japan, β2-adrenergic agonists, especially ritodrine hydrochloride, are often used for a prolonged period. At Ogaki Municipal Hospital, a tertiary care institution, asymptomatic pregnant women diagnosed with a short cervix were hospitalized and treated with prolonged intravenous ritodrine hydrochloride at the discretion of the attending physician until November 2015. However, a Cochrane review revealed that although β2-adrenergic agonists present efficacy in PTD reduction within 48 hours and 7 days of use, they do not have a preventive effect on PTD ^(14)^. The concentration of β2-receptors appears to gradually decrease with prolonged use of β2-adrenergic agonists, which leads to a form of desensitization ^(15)^, supporting only a short duration of action and use of these agents. Additionally, there are concerns regarding several severe side effects in both newborns and mothers, including neonatal hypoglycemia and hypocalcemia, maternal tachycardia, pulmonary edema, symptomatic arrhythmias, and (albeit rarely) myocardial infarction and maternal death, which prompted the US Food and Drug Administration to issue warnings against the use of β2-adrenergic agonists for PTD prevention ^(16)^. However, its administration was discontinued at our hospital due to concerns about the side effects associated with the long-term use of intravenous ritodrine hydrochloride. In August 2018, we were compelled to change our treatment approach, and vaginal progesterone treatment was initiated in all hospitalized pregnant women with CL shortening of ≤25 mm, based on the anticipation of potentially preventing PTD.

Thus, this study aims to evaluate the efficacy of vaginal progesterone in prolonging the interval from the diagnosis of a short cervix to delivery and in preventing PTD in asymptomatic women diagnosed with a short cervix after 24 weeks of gestation, using data from two cohort periods with varying treatment strategies. Furthermore, we evaluated neonatal outcomes associated with differences in periods (treatment strategies change), specifically focusing on neonatal intensive care unit (NICU) admissions, intubation, surfactant administration, intraventricular hemorrhage (IVH), and neonatal mortality.

## Materials and Methods

**Figure 1.**
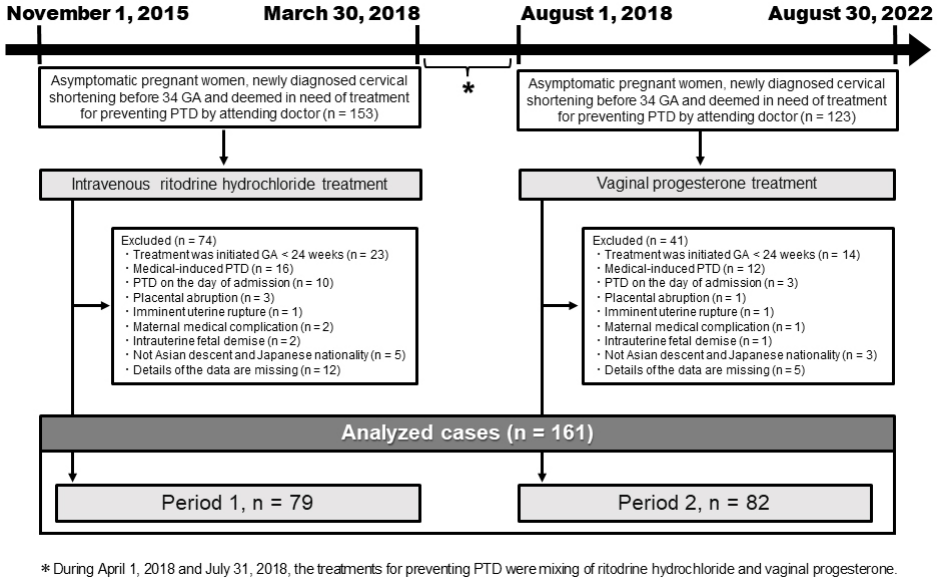
Flow diagram of study design. GA, gestational age; PTD, preterm delivery.

Patient enrollment and medical interventions were conducted at Ogaki Municipal Hospital in this historical cohort study. Subsequent analysis of outcomes was carried out at Nagoya University. Clinical data on maternal and neonatal characteristics was extracted from the hospital records. In Figure 1, during the period from November 2015 to March 2018 (Period 1), prolonged intravenous ritodrine hydrochloride was administered to 153 singleton pregnancies deemed in need of treatment for PTD prevention by the attending doctor. In April 2018, the Internal Ethical Committee approved off-label use of vaginal progesterone tablets. Ritodrine hydrochloride was unavailable in the hospital from August 2018 to August 2022 (Period 2). Thus, the treatment plan in Period 2 was changed; vaginal progesterone treatment for PTD prevention (200 mg, once daily) was formally implemented in asymptomatic women between 22 and 36 weeks of gestation and administered to 123 singleton pregnancies from the time of detection of the short cervix (equal indicates that hospital care was initiated). For the off-label use of vaginal progesterone tablets, all the women provided written informed consent prior to starting the treatment. In Period 1, on the basis of the discretion of the attending physician, some asymptomatic women with CL between 25 and 30 mm received prophylactic treatment with ritodrine hydrochloride. Nevertheless, in Period 2, stricter criteria were applied due to the off-label use of vaginal progesterone, and treatment was limited to asymptomatic women with CL < 25mm; the pregnant women with CL of 25-30 mm in Period 2 were managed with close observation, and progesterone treatment was initiated if their CL decreased to <25 mm.

The inclusion criteria were set as pregnant women with a newly diagnosed short cervix between 24 and 33 weeks of gestation. The women who were not included in the study were those who were urgently transported from other medical facilities because of short cervix or other symptoms. We also excluded the women who met any of the following criteria: received treatment before 24 weeks; had medically induced PTD, PTD on the day of admission, placental abruption, imminent uterine rupture, maternal complications, intrauterine fetal demise, and missing data; and were not of Asian descent and Japanese nationality. Consequently, the current study was carried out using data from 79 women in Period 1 and 82 women in Period 2, as in Figure 1.

The primary outcomes were PTD <37 weeks and <34 weeks, as well as NICU admission. The interval from the diagnosis of short cervix to delivery <14 days and <28 days, infant intubation, and surfactant administration were analyzed as secondary outcomes. Using SPSS version 28.0 for Windows (SPSS, Inc., Chicago, IL, USA), statistical analyses were carried out. Using the Mann-Whitney U test for continuous variables and Fisher’s exact test for categorical variables, each characteristic and perinatal outcomes were compared between Periods 1 and 2. To evaluate possible relationships with the outcomes, univariable and multivariable logistic regression analyses were performed using various characteristics; crude and adjusted odds ratios (ORs) and 95% confidence intervals (CIs) were reported as the OR (95% CI) for Period 2 (vaginal progesterone treatment), with Period 1 (intravenous ritodrine hydrochloride) utilized as the reference, and evaluated after adjusting for maternal age at delivery, parity, body mass index, infertility treatment, gestational age, CL at the start of maternal treatment, cervical cerclage, history of cervical conization, intravenous magnesium sulfate, oral calcium channel blockers, and histological chorioamnionitis. Statistical significance was set at *p* < 0.05.

Approval of the present study was provided by the Institutional Review Board of Ogaki Municipal Hospital and Nagoya University (approval number: 20150415), which also waived the requirement for written informed consent because of the retrospective nature of the study.

## Results

Table 1 shows the characteristics of the pregnant women in each period: 79 in Period 1 and 82 in Period 2. All eligible women in both groups were of Asian descent and Japanese nationality. All women in Period 1 were treated with intravenous ritodrine hydrochloride, whereas all women in Period 2 received the vaginal progesterone treatment. The administration of magnesium sulfate, antenatal steroid treatment, and cesarean section were carried out at the discretion of the attending physicians; in comparison with Period 1, Period 2 showed approximately 22% less magnesium sulfate administration, 17% less steroid treatment, and 15% less cesarean sections. Although in a very limited number of cases, calcium channel blockers have also been utilized to prevent PTD. Compared with Period 1, women in Period 2 commenced the treatment at an earlier gestational age, and the CL at the initiation of treatment was significantly shorter.

**Table 1.** Characteristics of the Study Groups.

	Period 1	Period 2	*p*
	November 2015-March 2018	August 2018-August 2022	
	(*n* = 79)	(*n* = 82)	
*Treatment for preventing and preparing preterm delivery*			
Ritodrine hydrochloride			
Intravenous	79 (100.0)	0 (0.0)	**<0.001**
Oral	9 (11.4)	0 (0.0)	**0.001**
Vaginal progesterone	0 (0.0)	82 (100.0)	**<0.001**
Intravenous magnesium sulphate	31 (39.2)	14 (17.1)	**0.003**
Oral calcium channel blockers	2 (2.5)	4 (4.9)	0.682
Antenatal steroid treatment	28 (35.4)	15 (18.3)	**0.020**
*Maternal and delivery characteristics*			
Maternal age (years)	32.0 (29.0-35.0)	32.5 (29.0-36.0)	0.723
Multiparity	46 (58.2)	45 (54.9)	0.751
Race (nationality): Asian (Japanese)	79 (100.0)	82 (100.0)	NA
Body mass index	20.3 (18.4-22.0)	20.2 (19.2-22.4)	0.431
Infertility treatment	9 (11.4)	20 (24.4)	**0.040**
GA at maternal treatment start (weeks)	30.9 (28.8-31.7)	28.9 (26.8-30.7)	**<0.001**
CL at maternal treatment start (mm)	22.0 (12.5-31.0)	16.0 (11.3-19.0)	**0.001**
Previous preterm delivery	12 (15.2)	14 (17.1)	0.832
Cervical cerclage	5 (6.3)	2 (2.4)	0.271
History of cervical conization	3 (3.8)	2 (2.4)	0.678
Cesarean section	36 (45.6)	25 (30.5)	0.053
Histological chorioamnionitis	25 (31.6)	19 (23.2)	0.289

Data are presented as medians (interquartile ranges) or *n* (%). GA: gestational age, CL: cervical length, NA: not applicable.

Table 2 shows the perinatal outcomes in each group. The gestational age at birth was significantly earlier in Period 1; consequently, the PTD rate (both <37 weeks) was higher. The interval from the diagnosis of short cervix to delivery was less than 14 days in 26 cases (32.9%) in Period 1 and seven cases (8.5%) in Period 2. The interval from the diagnosis of short cervix to delivery was less than 28 days in 30 cases (38.0%) in Period 1 and 11 cases (13.4%) in Period 2. In terms of PTD <34 weeks, there were 26 (32.9%) in Period 1 and 13 (15.9%) in Period 2. Expectedly, neonatal birth weight was lower in Period 1. In Period 2, a significant decrease in the proportion of infants requiring intubation was observed. Moreover, although not statistically significant, we observed a trend toward a reduction in cases that require NICU admission and surfactant administration. In this study group, there were few cases of neonatal death or neonatal IVH.

**Table 2.** Comparison of Perinatal Outcomes between the Study Groups.

	Period 1	Period 2	*p*
	November 2015-March 2018	August 2018-August 2022	
	(*n* = 79)	(*n* = 82)	
GA at birth (weeks)	36.6 (32.3-37.6)	38.2 (36.2-39.1)	**<0.001**
Preterm delivery (<37 weeks)	43 (54.4)	24 (29.3)	**0.001**
Preterm delivery (<34 weeks)	26 (32.9)	13 (15.9)	**0.016**
Interval from the diagnosis of short cervix to delivery <14 days	26 (32.9)	7 (8.5)	**<0.001**
Interval from the diagnosis of short cervix to delivery <28 days	30 (38.0)	11 (13.4)	**0.001**
Birth weight (kg)	2.43 (1.83-2.96)	2.85 (2.41-3.18)	**0.002**
Male	43 (54.4)	44 (53.7)	1.000
1 min Apgar score	8.0 (7.0-8.0)	8.0 (8.0-8.0)	0.480
5 min Apgar score	9.0 (8.0-9.0)	9.0 (8.3-9.0)	0.184
Umbilical artery pH <7.10	0 (0.0)	1 (1.2)	1.000
Umbilical artery BE <−10.0	0 (0.0)	3 (3.7)	0.246
NICU admission	39 (49.4)	32 (39.0)	0.207
Neonatal			
Intubation	14 (17.7)	5 (6.1)	**0.028**
Surfactant administration	11 (13.9)	4 (4.9)	0.060
Intraventricular hemorrhage	1 (1.3)	0 (0.0)	0.491
Death	0 (0.0)	1 (1.2)	1.000

Data are presented as medians (interquartile ranges) or *n* (%). GA: gestational age, CL: cervical length, NA: not applicable, NICU: neonatal intensive care unit.

Subsequently, we performed univariable and multivariable logistic regression analyses and identified several independent factors associated with each outcome (Table 3 and Supplemental Table 1). During Period 2, in which vaginal progesterone treatment was administered to all patients, a significant decrease was found in cases that lead to PTD (birth < 37 weeks) (adjusted OR 0.43, 95% CI 0.19-0.96) and PTD <14 days or 28 days following confirmation of short cervix (adjusted OR 0.21, 95% CI 0.06-0.72; adjusted OR 0.25, 95% CI 0.09-0.74, respectively). We conducted a post hoc power analysis for PTD (birth < 37 weeks) as the primary outcome, which yields a favorable result of 0.873, implying that the results of this study are statistically reliable and significant. We also observed that implementing progesterone treatment (Period 2) instead of ritodrine hydrochloride therapy (Period 1) was an independent factor in reducing the proportion of infants that require intubation (adjusted OR 0.17, 95% CI 0.04-0.75).

**Table 3.** Logistic Regression Analysis; Association between Periods 1 and 2 and Primary/Secondary Outcomes.

	Crude OR (CI)	Adjusted OR (CI)
Primary outcomes	
Preterm delivery (GA at birth <37 weeks)	**0.35 (0.18-0.66)**	**0.43 (0.19-0.96)**
Preterm delivery (GA at birth <34 weeks)	**0.38 (0.18-0.82)**	0.39 (0.13-1.21)
NICU admission	0.66 (0.35-1.23)	0.95 (0.42-2.15)
Secondary outcomes		
Extension of pregnancy <14 days	**0.19 (0.08-0.47)**	**0.21 (0.06-0.72)**
Extension of pregnancy <28 days	**0.25 (0.12-0.55)**	**0.25 (0.09-0.74)**
Neonatal Intubation	**0.30 (0.10-0.88)**	**0.17 (0.04-0.75)**
Neonatal surfactant administration	0.32 (0.10-1.04)	0.19 (0.03-1.14)

Each result is reported as the OR (95% CI) for Period 2, with Period 1 employed as the reference. Adjusted OR and 95% CI were evaluated after adjusting for maternal age (≥35 years) at delivery, multiparity, body mass index >25.0, infertility treatment, gestational age at maternal treatment start (intravenous ritodrine hydrochloride in Period 1 and vaginal progesterone treatment in Period 2), CL at the start of maternal treatment, cervical cerclage, history of cervical conization, intravenous magnesium sulfate, oral calcium channel blockers, and histological chorioamnionitis. GA, gestational age; OR, odds ratio; CI, confidence interval, CL, cervical length, NICU: neonatal intensive care unit.

**Figure 2.**
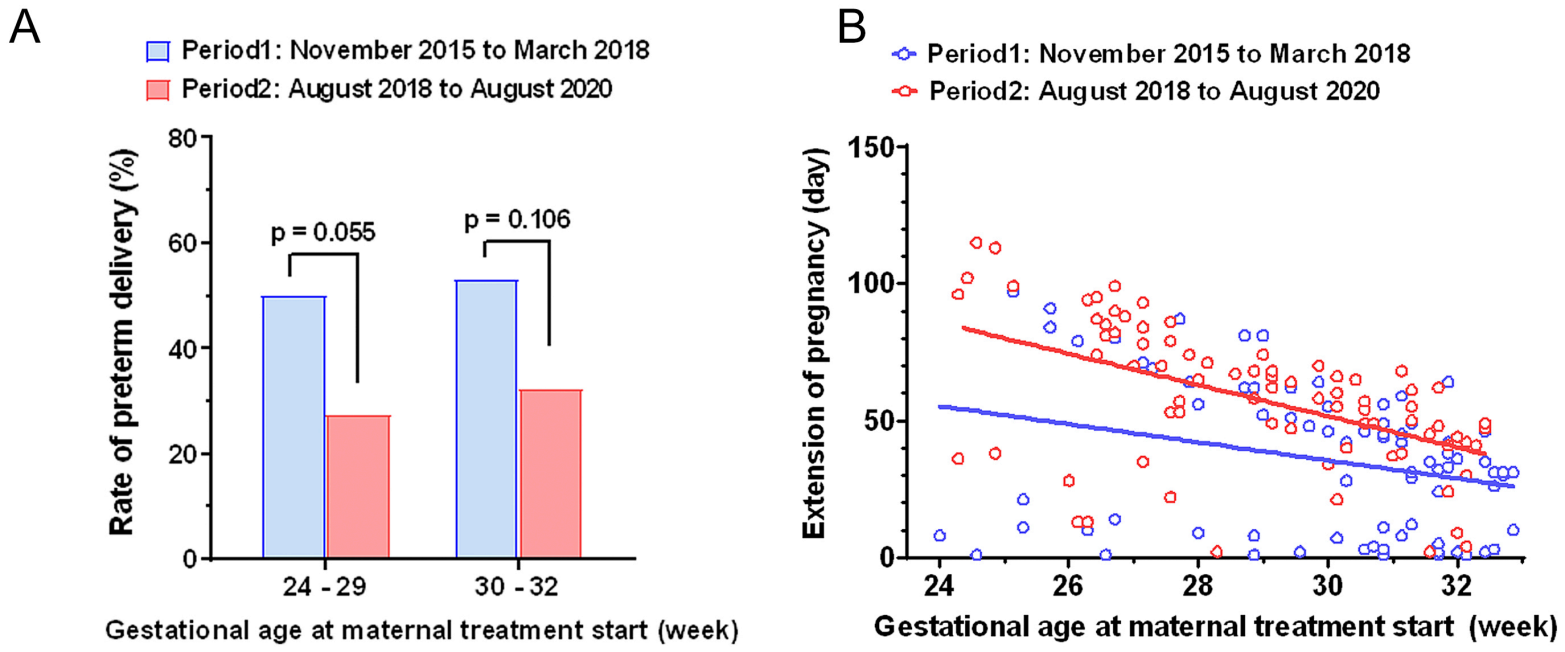
A) Comparison of preterm delivery rates in each period; 24-29 weeks or 30-32 weeks of gestation. The blue bar represents Period 1, and the red bar indicates Period 2. B) Scatterplots depicting the relationship between the number of days of pregnancy extension and the gestational age at which maternal treatment was initiated. The blue circles and line represent the values for each patient and best-fit line in Period 1, whereas the red circles and line indicate those in Period 2. A trend was observed in the correlation coefficients between the two groups, but it was not statistically significant (Fisher’s *z* transformation; *p* = 0.099).

The reduction in the PTD rate among women treated with vaginal progesterone can be examined in a subgroup analysis: At 24-29 weeks of gestation, the risk for PTD ranged from 50.0% (Period 1) to 27.5% (Period 2); at 30-32 weeks of gestation, the risk ranged from 53.1% to 32.3% (Figure 2A). Women who received progesterone treatment between 24 and 29 weeks show a trend toward a lower PTD rate than those who did not undergo the treatment (*p* = 0.055). Figure 2B presents scatterplots that depict the relationship between the number of days of pregnancy extension and the gestational age at which maternal treatment was initiated. The blue circles and lines represent the values for each patient and the best-fit line in Period 1, respectively, whereas the red circles and lines indicate those in Period 2. To compare the correlation coefficients between the two groups, we used Fisher’s *z* transformation. Although the result was not statistically significant, a trend was observed (*p* = 0.099). Overall, women treated with progesterone (Period 2) were observed to have a tendency to achieve a longer interval from the diagnosis of short cervix to delivery.

## Discussion

The current study first indicated that in a cohort of Japanese pregnant women with a short cervix, vaginal progesterone treatment significantly reduces PTD (birth < 37 weeks of gestation) and the PTD occurring within 14 or 28 days after confirming the short cervix, which confirms previous studies ^(11), (12), (13)^. We could not determine the effect of reducing the rate of NICU admission; nonetheless, our findings present a significant decrease in neonatal intubation. These findings suggest that in a Japanese clinical practice, vaginal progesterone treatment may be beneficial for asymptomatic singleton women with a short cervix newly diagnosed after 24 weeks of gestation.

There is a growing momentum in Japan to enhance the usage of β2-adrenergic agonists; thus, ritodrine hydrochloride, both orally and by injection, was not available during Period 2 of this study. Accordingly, we were able to conduct this historical cohort study. The most important advantage of this historical cohort design, in comparison with case-control design, is the mitigation of various types of bias risks. In particular, the strength of this study lies in the potential reduction of selection bias. The patients were not selected based on their risk and condition, and there was no room for the discretion of the attending physician; instead, the selection was based solely on the designated timeframe (Period 1 or 2). Furthermore, by collecting data from a single center, we were able to avoid the bias that comes from using a much different strategy for PTD prevention.

In 2022, the findings of an RCT, the TROPICAL study were reported ^(17)^. This is the only report from Japan that evaluated the efficacy of vaginal progesterone, and the results revealed that vaginal progesterone treatment significantly reduced the risk of PTD in singleton women with a mild short cervix (i.e., CL 25-30 mm) between 16 and 23 weeks of gestation. With persuasive evidence regarding the reduction in PTD risk in women with CL shortening before 24 weeks, the results of the TOPICAL study suggest the potential expansion of the indication for progesterone therapy in Japanese clinical practice. Nevertheless, the identification of short cervix after the late second trimester (i.e., ≥24 weeks of gestation) is highly relevant since most PTD occurs during this period ^(18)^. This raises the concern that the indication for vaginal progesterone should be expanded to women with a short cervix newly diagnosed after 24 weeks of gestation. Our findings are the first to address this point and suggest the benefit of expanding the indication of vaginal progesterone to Japanese women with a short cervix occurring during 24-33 weeks of pregnancy, especially at 24-29 weeks of gestation. Our study is valuable because numerous pregnant women at risk of PTD fall into the situation addressed in this study.

The retrospective nature of this study raises the possibility of several biases. This study lacked an established hospital protocol for detailed clinical management of PTD prevention. During both Periods 1 and 2, it was standard to measure CL once in the mid-trimester (e.g., 18-24 weeks). However, other aspects, such as the timing and frequency of additional CL measurements, criteria for diagnosing cervical incompetence, decisions on performing cerclage, adjustments in ritodrine infusion rates, criteria for administering steroids, and the use of additional tocolytics, were left to the discretion of the attending physician. This is a significant limitation of our study. Although the historical cohort study reduces the selection bias, due to the lack of randomization, the possibility of bias in patient background would still exist. Attention must be given to non-negligible differences among the present eligible women, including the rate of chorioamnionitis (CAM), cervical cerclage, and history of PTD. Therefore, in interpreting the present results, some caution should be exercised. Additionally, this study was carried out at a single center, which may limit the generalizability of the findings to a broader population. The findings should vary in different clinical settings and populations and require validation through prospective controlled trials with a large sample size. The final outcomes concerning the effectiveness of PTD prevention should include not only prolongation of the pregnancy but also perinatal morbidities such as long-term neurodevelopmental outcomes. The latter was not addressed in the current study and must be evaluated in the future.

In conclusion, this historical cohort study first suggests a possible beneficial efficacy of vaginal progesterone treatment; vaginal progesterone treatment may significantly reduce PTD and have the effect of prolonging gestational duration among Japanese pregnant women with a short cervix occurring between 24 and 33 weeks of gestation, which results in a reduction of infants requiring intubation. Although further validation through prospective controlled trials is warranted, these findings may contribute to expanding the use of vaginal progesterone treatment in Japanese clinical settings to enhance perinatal prognosis.

## Article Information

### Conflicts of Interest

None

### Acknowledgement

We thank Sachiko Morisaki for her valuable technical support and Editage (www.editage.jp) for the English language editing.

### Author Contributions

Naoki Otsuka and Kenji Imai contributed to the concept and design of the study and carried out the statistical analyses. Kenji Imai drafted the first version of the manuscript. Kenji Imai and Tomomi Kotani were involved in analyzing and interpreting the data. Takafumi Ushida, Seiko Matsuo, Sho Tano, Masataka Nomoto, Yukako Iitani, Mika Ishi, Yosuke Kawai, Toshimitsu Furui, and Hiroaki Kajiyama contributed to the interpretation of data and provided critical feedback throughout the preparation of the manuscript. Naoki Otsuka and Kenji Imai critically reviewed the manuscript. The final version of the manuscript was approved by all of the authors.

### Approval by Institutional Review Board (IRB)

Approval of the present study was provided by the Institutional Review Board of Ogaki Municipal Hospital and Nagoya University (approval number: 20150415).

## Supplement

Supplemental Table 1

## References

[1] Blencowe H, Cousens S, Chou D, et al. Born too soon: the global epidemiology of 15 million preterm births. Reprod Health. 2013;10(Suppl 1):S2. 24625129 10.1186/1742-4755-10-S1-S2 PMC3828585

[2] Niwa Y, Imai K, Kotani T, et al. Relationship between cytokine profiles of cord blood and cord S100B levels in preterm infants. Early Hum Dev. 2019;129:65-70. 30684905 10.1016/j.earlhumdev.2019.01.013

[3] Ohori Y, Imai K, Tano S, et al. Predicting preterm birth within 2 weeks in asymptomatic women with a short cervix: combined effects of cervicovaginal fluid cytokine levels and fetal fibronectin test. J Obstet Gynaecol Res. 2024;50(4):587-95. 38217336 10.1111/jog.15889

[4] Berghella V, Lesser T, Boelig RC, et al. Cervical length screening after 24 weeks for prediction and prevention of preterm birth: not evidence based yet. Am J Obstet Gynecol MFM. 2020;2(2):100097. 33345963 10.1016/j.ajogmf.2020.100097

[5] Crane JM, Hutchens D. Transvaginal sonographic measurement of cervical length to predict preterm birth in asymptomatic women at increased risk: a systematic review. Ultrasound Obstet Gynecol. 2008;31(5):579-87. 18412093 10.1002/uog.5323

[6] Papastefanou I, Pilalis A, Eleftheriades M, et al. Prediction of preterm delivery by late cervical length measurement after 24 weeks. Fetal Diagn Ther. 2015;38(3):200-4. 26367859 10.1159/000381144

[7] Coutinho CM, Sotiriadis A, Odibo A, et al. ISUOG Practice Guidelines: role of ultrasound in the prediction of spontaneous preterm birth. Ultrasound Obstet Gynecol. 2022;60(3):435-56. 35904371 10.1002/uog.26020

[8] Hassan SS, Romero R, Vidyadhari D, et al. Vaginal progesterone reduces the rate of preterm birth in women with a sonographic short cervix: a multicenter, randomized, double-blind, placebo-controlled trial. Ultrasound Obstet Gynecol. 2011;38(1):18-31. 21472815 10.1002/uog.9017 PMC3482512

[9] Romero R, Nicolaides KH, Conde-Agudelo A, et al. Vaginal progesterone decreases preterm birth < /= 34 weeks of gestation in women with a singleton pregnancy and a short cervix: an updated meta-analysis including data from the OPPTIMUM study. Ultrasound Obstet Gynecol. 2016;48(3):308-17. 27444208 10.1002/uog.15953 PMC5053235

[10] Romero R, Conde-Agudelo A, Da Fonseca E, et al. Vaginal progesterone for preventing preterm birth and adverse perinatal outcomes in singleton gestations with a short cervix: a meta-analysis of individual patient data. Am J Obstet Gynecol. 2018;218(2):161-80. 29157866 10.1016/j.ajog.2017.11.576 PMC5987201

[11] Azargoon A, Ghorbani R, Aslebahar F. Vaginal progesterone on the prevention of preterm birth and neonatal complications in high risk women: a randomized placebo-controlled double-blind study. Int J Reprod Biomed. 2016;14(5):309-16. 27326415 PMC4910039

[12] Cetingoz E, Cam C, Sakalli M, et al. Progesterone effects on preterm birth in high-risk pregnancies: a randomized placebo-controlled trial. Arch Gynecol Obstet. 2011;283(3):423-9. 20091317 10.1007/s00404-009-1351-2

[13] Fonseca EB, Celik E, Parra M, et al. Progesterone and the risk of preterm birth among women with a short cervix. N Engl J Med. 2007;357(5):462-9. 17671254 10.1056/NEJMoa067815

[14] Neilson JP, West HM, Dowswell T. Betamimetics for inhibiting preterm labour. Cochrane Database Syst Rev. 2014;2014(2):CD004352. 24500892 10.1002/14651858.CD004352.pub3 PMC10603219

[15] Berg G, Andersson RG, Ryden G. Beta-adrenergic receptors in human myometrium during pregnancy: changes in the number of receptors after beta-mimetic treatment. Am J Obstet Gynecol. 1985;151(3):392-6. 2982269 10.1016/0002-9378(85)90310-2

[16] Haas DM, Benjamin T, Sawyer R, et al. Short-term tocolytics for preterm delivery - current perspectives. Int J Womens Health. 2014;6:343-9. 24707187 10.2147/IJWH.S44048 PMC3971910

[17] Hayashi M, Oi R, Otsuki K, et al. Effects of prophylactic vaginal progesterone administration on mild cervical shortening (TROPICAL study): a multicenter, double-blind, randomized trial. J Matern Fetal Neonatal Med. 2022;35(25):8012-8. 34182873 10.1080/14767058.2021.1940935

[18] Shapiro-Mendoza CK, Lackritz EM. Epidemiology of late and moderate preterm birth. Semin Fetal Neonatal Med. 2012;17(3):120-5. 22264582 10.1016/j.siny.2012.01.007 PMC4544710

