# Supplementary material for: Possible Efficacy of Vaginal Progesterone on Asymptomatic Women with a Short Cervix after 24 Weeks of Gestation: A Historical Cohort Study in Japan
Source: JMA J. 2024 Sep 20;7(4):582–9. doi: 10.31662/jmaj.2024-0036 (PMC11543341; doi:10.31662/jmaj.2024-0036)
Supplement: Supplemental Table 1 [file 2433-3298-7-4-0582-s001.pdf]

Supplemental Table 1. Logistic regression analysis; Association of each characteristic to the perinatal outcomes.

	Preterm delivery (GA at birth < 37 weeks)	Interval from the diagnosis of short cervix to delivery < 14 days	Interval from the diagnosis of short cervix to delivery < 28 days	Neonatal intubation
	Adjusted OR (CI)			
Maternal age (≥ 35 years)	0.53 (0.21 to 1.35)	0.36 (0.10 to 1.31)	0.49 (0.15 to 1.59)	0.81 (0.20 to 3.32)
Multiparity	0.77 (0.33 to 1.79)	1.71 (0.55 to 5.37)	1.08 (0.38 to 3.06)	1.54 (0.37 to 6.41)
Body mass index > 25.0	0.73 (0.21 to 2.61)	0.70 (0.07 to 6.98)	0.50 (0.06 to 4.43)	0.37 (0.03 to 4.34)
Infertility treatment	1.22 (0.42 to 3.52)	2.33 (0.57 to 9.64)	1.77 (0.49 to 6.43)	4.75 (0.94 to 23.9)
GA at maternal treatment start (weeks)	1.10 (0.93 to 1.31)	1.01 (0.81 to 1.26)	1.07 (0.87 to 1.32)	**0.58 (0.44 to 0.78)**
CL at maternal treatment start (≤ 15 mm)	1.88 (0.83 to 4.24)	0.73 (0.23 to 2.32)	1.79 (0.64 to 4.99)	0.37 (0.08 to 1.62)
Cervical cerclage	0.55 (0.08 to 3.55)	0.88 (0.11 to 7.10)	1.52 (0.22 to 10.7)	2.53 (0.23 to 27.4)
History of cervical conization	2.49 (0.24 to 25.5)	**16.6 (1.03 to 269)**	10.9 (0.84 to 141)	10.3 (0.91 to 117)
Intravenous magnesium sulphate	**6.15 (2.46 to 15.4)**	**10.3 (3.51 to 29.9)**	**7.71 (2.91 to 20.4)**	**6.04 (1.53 to 23.9)**
Oral calcium channel blockers	2.91 (0.38 to 22.4)	7.44 (0.75 to 74.1)	**13.2 (1.40 to 125)**	1.71 (0.11 to 0.26)
Vaginal progesterone	**0.43 (0.19 to 0.96)**	**0.21 (0.06 to 0.72)**	**0.25 (0.09 to 0.74)**	**0.17 (0.04 to 0.75)**
Histological chorioamnionitis	**3.48 (1.41 to 8.56)**	**3.16 (1.08 to 9.29)**	**4.04 (1.46 to 125)**	1.99 (0.51 to 7.67)

Results are reported as OR (95% CI). GA, gestational age; CL, cervical length; PTD, preterm delivery; OR, odds ratio; CI, confidence interval.
